# Analysis of Changes in Traumatic Symptoms and Daily Life Activity of Children Affected by the 2011 Japan Earthquake and Tsunami over Time

**DOI:** 10.1371/journal.pone.0088885

**Published:** 2014-02-19

**Authors:** Masahide Usami, Yoshitaka Iwadare, Kyota Watanabe, Masaki Kodaira, Hirokage Ushijima, Tetsuya Tanaka, Maiko Harada, Hiromi Tanaka, Yoshinori Sasaki, Kazuhiko Saito

**Affiliations:** 1 Department of Child and Adolescent Psychiatry, National Center for Global Health and Medicine, Kohnodai Hospital, Chiba, Japan; 2 Department of Child Mental Health, Imperial Gift Foundation, Aiiku Hospital, Tokyo, Japan; University of Sao Paulo, Brazil

## Abstract

**Background:**

On March 11, 2011, Japan was struck by a massive earthquake and tsunami. The tsunami caused tremendous damage and traumatized a number of people, including children. This study aimed to compare traumatic symptoms and daily life activity among children 20 months after the 2011 Great East Japan Earthquake and Tsunami with those observed after 8 months.

**Methods:**

The study comprised two groups. The first comprised 12,524 kindergarten, elementary school, and junior high school children in Ishinomaki City, Miyagi Prefecture, Japan, who were evaluated 8 months after the disaster. The second comprised 10,597 children from the same place who were evaluated 20 months after the disaster. The Post Traumatic Stress Symptoms for Children 15 items (PTSSC-15), a self-completion questionnaire on traumatic symptoms, and a questionnaire on children's daily life were distributed to the children. An effective response was obtained from 11,639 (92.9%, 8 months after) and 10,597 (86.9%, 20 months after) children.

**Results:**

The PTSSC-15 score was significantly higher in junior high school girls than in boys. The PTSSC-15 score was significantly higher in 4th–6th grade girls than in boys after 8 months. Elementary and junior high school children evaluated after 20 months had a significantly lower PTSSC-15 score than those evaluated after 8 months. The number of children having breakfast was significantly higher after 8 months than that after 20 months. In both the groups, children of all grades who had breakfast had a significantly lower PTSSC-15 score than those who did not have breakfast.

**Conclusions:**

We conclude that traumatic symptoms and daily life activity of children who survived the earthquake and tsunami improved over time.

## Background

On March 11, 2011, Japan was struck by a massive earthquake and tsunami. The tsunami caused tremendous damage and traumatized a number of people, including children[1]–[8]. Till date, a number of studies have been conducted on children who have survived disasters [7]–[20].

After a disaster, posttraumatic stress disorder (PTSD) is the psychiatric diagnosis that should be considered most carefully[11], [21], [22]. However, traumatic symptoms tend to spontaneously heal over time; therefore, the morbidity of PTSD is dependent on the time, subjects, and methods used in the survey[12], [14], [23]–[29].

We collected information on daily life activity, environmental damage conditions, and traumatic symptoms of children who survived the 2011 Japanese Earthquake and Tsunami 8 months after the disaster. That study demonstrated that relationships between environmental damage conditions and traumatic symptoms were dependent on gender, age, house damage, evacuation experience, and bereavement experience[7]. Furthermore, children with house damage or evacuation experiences slept for a significantly shorter duration than those without these experiences[8].

Twenty months following the disaster, all shelters were closed, and the reconstruction, donations, and installation of temporary housing are still progressing. It is necessary to discuss whether traumatic symptoms and daily life activity of the children who survived the disaster has improved 20 months after the disaster in comparison with those after 8 months. Therefore, we collected information on the traumatic symptoms and daily life activity of these children 20 months after the disaster.

This study aimed to evaluate and compare the changes in traumatic symptoms of children 8 and 20 months after the earthquake and tsunami. The main hypothesis of this study was that traumatic symptoms and daily life activities of children who survived the tsunami improved between 8 and 20 months after the disaster. This hypothesis indicates that these children live under stable life environments such as going to school, having breakfast daily, and retaining a comfortable sleep environment in their new homes.

## Methods

Our Survey was conducted according to the principles expressed in the Declaration of Helsinki.

Each survey was conducted as a part of the school education program under the initiative of the Board of Education in Ishinomaki City.

Ishinomaki City is the second largest city (population, 162,822) in Miyagi Prefecture, Japan. As of February 15, 2012, the death toll in Ishinomaki City was 3,182, and 557 people were missing. The total number of collapsed houses and buildings, including half-collapsed houses, was 33,378, and 7,298 temporary houses had been constructed.

Surveys were distributed to all children who attended five kindergartens, 43 elementary schools, and 21 junior high schools in Ishinomaki City, Miyagi Prefecture. The survey was conducted in November 2011 and 2012 (8 and 20 months after the earthquake disaster, respectively). Some children who survived the huge tsunami had migrated to another city from Ishinomaki city. The number of children who went to municipal kindergartens, elementary schools, and junior high schools were 12534 in November 2011, and the number was 12193 in November 2012.

The method of administering surveys in 2011 and 2012 was the same. First, the Education Committee of Ishinomaki City explained the survey method to the principals of all of the schools. Following this, the teachers distributed a letter explaining the survey, which had been constructed by the Education Committee, to all children and their parents. The letter clearly stated that by filling the questionnaire, both student and parents were considered to be providing consent to participate in the survey. The letter also specified that the survey results would be used to provide children with psychological care to facilitate their education at the school and that the results would be published as a medical paper. Informed consent was thus obtained when the students filled the questionnaire. The Ethical Committee of the National Center for Global Health and Medicine approved this study, including the consent procedure.

### Participants

On November 2011 (8 months after the disaster), PTSSC-15, a self-completion questionnaire on traumatic symptoms, was distributed to 12,524 children registered at municipal schools in Ishinomaki City. A self-completion questionnaire about the daily life, waking time, time of sleep onset, and breakfast consumption was also distributed to 12, 534 children. A questionnaire on the environmental damage experienced by the children was distributed to their teachers. On November 2012 (20 months after the disaster), PTSSC-15 and the questionnaire on daily life were distributed to 12,193 children registered at municipal schools and to their teachers.

Parents of kindergartners and 1st–3rd grade elementary school students were asked to fill the questionnaire while talking to their children. Informed consent for participation in the survey was obtained when the completed questionnaires were received from the children.

Answers were obtained from 12,346 (98.6%) of the 12,524 children (8 months after) and 11,124 (91.2%) of the 12,193 children (20 months after) to whom questionnaires were distributed. An effective response was obtained from 11,639 (92.9%, 8 months after) and 10,597 (86.9%, 20 months after) children. Effective responses at 20 months had no connection with effective responses at 8 months by anonymity.

Answers to the questionnaire on environmental damage 8 months after the disaster with regard to all 12,524 children were obtained from teachers. Table 1 shows data regarding gender, age, and environmental damage conditions (house damage, evacuation conditions, and bereavement experience) in 11,639 children 8 months after the disaster (Table 1)[6], [7]. When teachers had no information regarding house damage, evacuation conditions, and bereavement experience, the answer was defined as “unknown.”

**Table 1:** Damage condition of children affected by the 2011 Japan Earthquake and Tsunami.

Items			N = 11639	
House damage	No		6986	(60.0%)
	Yes	Total collapse	2243	(19.3%)
		Half collapse	2354	(20.2%)
		Total	4597	(39.5%)
	Unknown		56	(0.5%)
Evacuation experience	No		8228	(70.7%)
	Yes	Currently living in an evacuation center	90	(0.8%)
		Used to live in an evacuation center	2845	(24.4%)
		Living in temporary housing	976	(8.4%)
		Used to live in temporary housing	51	(0.4%)
		Evacuation experience at least once	3248	(27.9%)
	Unknown		163	(1.4%)
Bereavement experience	No		9241	(79.4%)
	Yes	Father	71	(0.6%)
		Mother	66	(0.6%)
		Brothers and sisters	44	(0.4%)
		Grandfather and grandmother	355	(3.1%)
		Classmates	1498	(12.9%)
		Teacher in-charge	32	(0.3%)
		Others	270	(2.3%)
		At least one bereavement experience	2103	(18.1%)
	Unknown		295	(2.5%)

N, number of cases.

### Measures

This was a paper-based survey with questions regarding traumatic symptoms using a self-report form. The self-report form consisted of PTSSC-15 and a questionnaire on daily life developed by the authors.

### PTSSC-15

PTSSC-15 is a self-completion questionnaire on the stress reactions in children after disasters. A similar survey, the Posttraumatic Stress Symptom 10 (PTSS-10)[30], had fewer questions and was used as a screening test after the Great Hanshin Earthquake(Kato et al., 1996). PTSS-10 was administered to 105 Norwegian children (6–17 years old) 10 and 30 months after the 2004 South East Asia Tsunami[31]. Five questions that were considered to reveal important psychosomatic characteristics following disasters (flashback, appetite loss, somatic reactions such as headache and abdominal pain, attention deficit, and anxiety) were added to PTSS-10, and PTSSC-15 consisting of 15 questions was constructed in Japan[32].

Each question in the questionnaire is scored in six levels: 0 =  completely disagree, 1 =  mostly disagree, 2 =  partially disagree, 3 =  partially agree, 4 =  mostly agree, and 5 =  completely agree. Higher scores indicate more severe traumatic symptoms and depressive symptoms. Tominaga and colleagues demonstrated the reliability and validity of PTSSC-15 for Japanese children and adolescents[32].

### Questionnaire on daily life

The daily life questionnaire included items related to waking and sleep-onset times. For the total sleep duration on weekdays and holidays, the subject was asked to write the total usual daily sleep hours, usual wake-up time, and the time at which the subject usually goes to sleep.

The daily life questionnaire included items related to having breakfast every morning. Each question was scored in two levels: 0 =  did not usually have breakfast daily and 1 =  usually have breakfast daily.

## Statistical analysis

### PTSSC-15, school grades, and gender

Children were divided into four grade groups: kindergartners, 1st–3rd grade (elementary school students), 4th–6th grade (elementary school students), and 7th–9th grade (junior high school students). For grade group and gender, the median PTSSC-15 score and interquartile range were separately determined 8 and 20 months after the disaster. The PTSSC-15 score was statistically compared between males and females by the Mann–Whitney U test for each grade group. Effect sizes were then calculated on the basis of the Mann–Whitney statistics.

### Sleep duration and breakfast consumption

The average sleep duration on weekdays and holidays was also calculated for each grade and gender separately after 8 and 20 months. For children suffering from traumatic experiences, the average sleep duration on weekdays and holidays after 8 months was compared with that after 20 months.

The number of children who had breakfast after 8 and 20 months was calculated for each grade and gender. The number of children who had breakfast daily after 8 months was compared with that after 20 months for each grade and gender. The PTSSC-15 scores of children who usually had breakfast daily and those who did not usually have breakfast daily were separately compared after 8 and 20 months.

### Change in PTSSC-15 scores and daily life activities

The average PTSSC-15 scores in each grade group and gender was separately calculated after 8 and 20 months.

The difference in the average PTSSC-15 score after 8 and 20 months was statistically analyzed by two-factor analysis of variance for each grade group and gender.

The difference in the sleep duration after 8 and 20 months was statistically analyzed by two-factor analysis of variance for each gender and grade group.

The difference in the average PTSSC-15 score of those who had breakfast and those who did not was separately analyzed by two-factor analysis of variance for each gender and grade group after 8 and 20 months.

In all tests, a significance level of 0.05 was used in two-sided tests. Analyses were performed using PASW 18.0.

## Results

### Descriptive information

The participants evaluated 8 months after the disaster included 11,692 children (5959 males, 5733 females), while those evaluated after 20 months included 10749 children (5402 males, 5347 females) who were exposed to the 2011 Japanese Earthquake and Tsunami. Table 2 shows the gender, The Post Traumatic Stress Symptoms for Children 15 items (PTSSC-15) score, age, sleep duration, and breakfast consumption (Table 2).

**Table 2:** Characteristics of children affected by the 2011 Japan Earthquake and Tsunami.

Items			After 8 months			After 20 months	
			N = 11,639			N = 10,597	
Gender	Male		5939		(51.0%)	5302	(50.0%)
	Female		5700		(49.0%)	5295	(50.0%)
Age at the time of the disaster (y) (Mean)			10.9		(SD = 2.7)	10.9	(SD = 2.7)
PTSSC-15 score (Mean)			20.5		(SD = 14.5)	18.8	(SD = 14.0)
Sleep duration on weekdays (Mean)			8.4(h)		(SD = 1.3)	8.5(h)	(SD = 1.2)
Sleep duration on holidays (Mean)			9.0(h)		(SD = 1.4)	9.2 (h)	(SD = 1.3)
Breakfast consumption		yes	10673		(91.7%)	9928	(93.7%)
		no	827		(7.1%)	645	(6.1%)
		unknown	139		(1.2%)	24	(0.2%)

### PTSSC-15 score after 8 and 20 months

Table 3 shows the PTSSC-15 score after 8 and 20 months in each grade group and gender (Table 3). The PTSSC-15 score was significantly higher (P<0.001) in girls than in boys in the 7th–9th grade (junior high schools). The PTSSC-15 score was significantly higher (P<0.001) in girls than in boys in the 4th–6th grade after 8 months. These effect sizes were under 0.30.

**Table 3:** Average PTSSC-15 score (grade and gender).

		Gender												
		Male						Female						
		M	IR			N		M	IR			N	Effect size	P value
After 8 months	Kindergartners	14.0	3.0	–	25.5	119		15.0	3.0	–	25.5	127	0.03	ns
	1st–3rd grade	16.0	6.0	–	28.0	1866		17.0	7.0	–	28.0	1736	0.03	ns
	4th–6th grade	17.0	7.0	–	29.0	1975		20.0	9.0	–	32.0	1973	0.08	<0.0001
	7th–9th grade	20.0	9.0	–	32.0	1979		26.0	14.0	–	37.0	1864	0.15	<0.0001
After 20 months	Kindergartners	12.0	2.0	–	25.0	111		12.0	3.0	–	23.0	127	0.09	ns
	1st–3rd grade	15.0	5.0	–	26.0	1632		16.0	6.0	–	27.0	1581	0.03	ns
	4th–6th grade	15.0	6.0	–	28.0	1792		17.0	7.0	–	28.0	1798	0.05	ns
	7th–9th grade	18.0	8.0	–	29.0	1767		23.0	12.0	–	34.0	1789	0.29	<0.0001

M, median; IR, interquartile range; N, number of cases.

The average PTSSC-15 score was compared in each grade group, gender, and month (Table 4). Children in all grade groups, except kindergartners evaluated after 20 months, had a significantly lower PTSSC-15 score than children evaluated at 8 months [F(16811) = 14.97, P<0.0001; F(17534) = 53.20, P<0.0001; F(17395) = 35.57, P<0.0001].

**Table 4:** Average PTSSC-15 score (grade, gender, and month)

Grade group	Gender	Months after disaster								F	P value
		After 8 months				After 20 months					
		M	SD	N		M	SD	N			
Kindergartners									Gender × months	0.031	ns
	Male	15.1	12.5	119		13.2	11.6	111	Months	2.274	ns
	Female	15.7	12.8	127		14.2	12.5	127	Gender	0.504	ns
1st–3rd grade									Gender × months	0.416	ns
	Male	17.4	12.8	1866		16.4	12.9	1632	Months	14.97	<0.0001
	Female	18.2	12.8	1736		16.8	12.6	1581	Gender	3.741	ns
4th–6th grade									Gender × months	4.526	<0.05
	Male	19.6	14.4	1975		17.9	13.9	1792	Months	53.20	<0.0001
	Female	21.7	14.7	1973		18.6	14.0	1798	Gender	18.10	<0.0001
7th–9th grade									Gender × months	0.529	<0.0001
	Male	21.3	14.7	1979		19.5	14.0	1767	Months	35.57	<0.0001
	Female	26.0	15.5	1864		23.7	14.8	1789	Gender	167.6	<0.0001

M, mean; SD, standard deviation; N, number of cases.

### Sleep duration after 8 and 20 months

The average sleep duration on weekdays and holidays was compared in each grade group, gender, and month (Table 5). On weekdays, children in the 4th–6th grade group evaluated after 20 months had a significantly longer sleep duration than those evaluated after 8 months [F(17534) = 6.484, P<0.05]. On holidays, children in the 4th–9th grade group evaluated after 20 months had a significantly longer sleep duration than those evaluated after 8 months [(F(16811) = 5.533, P<0.05; F(17534) = 39.57, P<0.0001; F(17395) = 8.155, P<0.001).

**Table:** **Table5.** Average sleep duration on weekdays and holidays (grade, gender, and month).

	Grade group	Gender	Months after disaster								F	P value
			After 8 months				After 20 months					
			M	SD	N		M	SD	N			
Weekday	Kindergartners									Gender × months	2.122	ns
		Male	9.8	0.7	119		9.8	0.9	111	Months	2.122	ns
		Female	10.0	0.6	127		9.8	0.8	127	Gender	2.122	ns
	1st–3rd grade									Gender × months	0.0	ns
		Male	9.3	0.6	1866		9.3	0.7	1632	Months	0.0	ns
		Female	9.3	0.6	1736		9.3	0.6	1581	Gender	0.0	ns
	4th–6th grade									Gender × months	6.484	<0.05
		Male	8.7	0.9	1975		8.8	0.9	1792	Months	6.484	<0.05
		Female	8.7	0.8	1973		8.7	0.8	1798	Gender	6.484	<0.05
	7th–9th grade									Gender × months	0.0	ns
		Male	7.6	1.2	1979		7.6	1.2	1767	Months	0.0	ns
		Female	7.3	1.1	1864		7.3	1.0	1789	Gender	130.2	<0.0001
Holidays	Kindergartners									Gender × months	1.885	ns
		Male	9.9	0.8	119		9.9	0.8	111	Months	1.885	ns
		Female	10.2	0.8	127		10.0	0.8	127	Gender	7.539	<0.01
	1st–3rd grade									Gender × months	5.533	<0.05
		Male	9.3	0.9	1866		9.4	0.9	1632	Months	5.533	<0.05
		Female	9.7	0.8	1736		9.7	0.9	1581	Gender	271.1	<0.0001
	4th–6th grade									Gender × months	9.891	<0.001
		Male	8.6	1.5	1975		8.9	1.4	1792	Months	39.57	<0.0001
		Female	9.4	1.3	1973		9.5	1.3	1798	Gender	484.7	<0.0001
	7th–9th grade									Gender × months	1.85E-10	ns
		Male	8.5	1.6	1979		8.6	1.6	1767	Months	8.155	<0.001
		Female	8.8	1.4	1864		8.9	1.4	1789	Gender	73.40	<0.0001

M, mean; SD, standard deviation; N, number of cases.

### Breakfast consumption after 8 and 20 months

The number of children who had breakfast (10673 children, 91.7%) was greater after 8 months than that (9928 children, 93.7%) after 20 months (chi-square test, P<0.001).

The average PTSSC-15 scores of children who had and those who did not have breakfast were compared in each grade group, gender, and separately after 8 and 20 months (Table 6). After 8 months, children in all grade groups who had breakfast had a significantly lower PTSSC-15 score than those who did not have breakfast [F(1235) = 10.19, P<0.0001; F(13501) = 27.85, P<0.0001; F(13929) = 73.31, P<0.0001, F(13819) = 60.94, P<0.0001). After 20 months, children in all grade groups who had breakfast had a significantly lower PTSSC-15 score than those who did not have breakfast [F(1231) = 4.976, P<0.05; F(13206) = 25.75, P<0.0001; F(13576) = 36.07, P<0.0001, F(13544) = 27.47, P<0.0001].

**Table 6:** Average PTSSC-15 score (grade, gender, and breakfast consumption) in 2011 and 2012.

	Grade group	Gender	Eating breakfast								F	P value
			Yes				No					
			M	SD	N		M	SD	N			
2011	Kindergartners									Gender × breakfast consumption	0.4711	ns
		Male	14.1	11.5	110		35.4	13.5	5	Breakfast consumption	4.976	<0.05
		Female	15.3	12.7	120		20.5	10.1	4	Gender	0.073	ns
	1st–3rd grade									Gender × breakfast consumption	1.217	ns
		Male	17.1	12.6	1741		22.5	13.8	79	Breakfast consumption	25.75	<0.0001
		Female	18.0	12.7	1616		23.9	14.7	69	Gender	0.438	ns
	4th–6th grade									Gender × breakfast consumption	0.520	ns
		Male	18.9	13.9	1826		26.3	17.2	142	Breakfast consumption	36.07	<0.0001
		Female	21.1	14.5	1870		30.5	15.9	95	Gender	1.942	ns
	7th–9th grade									Gender × breakfast consumption	0.560	ns
		Male	20.9	14.7	1725		24.6	15.7	244	Breakfast consumption	27.47	<0.0001
		Female	24.9	15.2	1665		33.3	16.5	189	Gender	18.66	<0.0001
2012	Kindergartners									Gender × breakfast consumption	0.4711	ns
		Male	12.4	11.3	105		24.3	9.8	4	Breakfast consumption	4.976	<0.05
		Female	14.1	12.6	121		20.4	7.9	5	Gender	0.073	ns
	1st–3rd grade									Gender × breakfast consumption	1.217	ns
		Male	16.1	12.6	1554		23.1	14.9	77	Breakfast consumption	25.75	<0.0001
		Female	16.6	12.5	1523		21.1	14.2	56	Gender	0.438	ns
	4th–6th grade									Gender × breakfast consumption	0.520	ns
		Male	17.5	13.6	1678		23.0	16.2	108	Breakfast consumption	36.07	<0.0001
		Female	18.2	13.8	1712		25.2	15.8	82	Gender	1.942	ns
	7th–9th grade									Gender × breakfast consumption	0.560	ns
		Male	19.0	13.6	1568		24.2	15.9	192	Breakfast consumption	27.47	<0.0001
		Female	23.4	14.6	1667		27.3	16.6	121	Gender	18.66	<0.0001

M, mean; SD, standard deviation; N, number of cases.

## Discussion

This study showed that the traumatic symptoms and daily life activity of children who suffered damage and loss improved 20 months after this severe natural disaster compared with the traumatic symptoms and daily life activity of children evaluated 8 months after the disaster.

A previous study elucidated that PTSSC-15 scores were related to the environmental damage caused by the 2011 Japanese Tsunami^1^. This study showed that PTSSC-15 decreased over time. It suggested that children suffering traumatic symptoms from disasters such as the massive tsunami may recover over time. However, using only a self-completion questionnaire as a screening tool for PTSD after a huge disaster may result in an inflated number of children who appear to be at a high risk for PTSD. If psychiatrists or clinical psychologists use a self-completion questionnaire as a screening tool for PTSD after a disaster, they are obligated to treat a number of children detected with a high risk for PTSD[7], [34]. Therefore, a self-completion questionnaire is insufficient as a screening tool for PTSD after a disaster.

Differences in traumatic symptoms due to gender and age were recognized in this study. According to the effect size, the difference due to gender was negligible; however, the difference due to age was large. The responders in the case of kindergarten and lower-grade elementary school students were the parents, and it is possible that not only the status of the children but also the psychological anxiety and status of their parents affected the responses. Therefore, it is necessary to comprehensively judge the status of children by considering not only their psychological status but also the psychological conditions of their parents.

We found that the sleep duration on holidays in 1st–9th grade group after 20 months was longer than that after 8 months. The sleep duration on weekdays in the 4th–6th grade group after 20 months was longer than that after 8 months. A previous study elucidated that the relationship between sleep duration and traumatic symptoms displayed low correlations[8]. Thus, sleep duration may not be a good predictor of improvedtraumatic symptoms.

Furthermore, we found that having breakfast was closely related to traumatic symptoms of the children. These results suggest that parents may not be able to afford a good living environment following the tsunami and that the children were severely depressed and/or neglected. After the huge disaster, child abuse, orphans, and poverty are a serious issue in Japan[33], [34]. Thus, for children with traumatic symptoms, greater support of the family may be important. This segment of the questionnaire regarding having breakfast daily may be useful for quick screening of children with traumatic symptoms or parents with a reduced family support function.

### Limitations

This study was an observation study that compared the same age group at 8 months and at 20 months despite the fact that those examined at 8 months were a year older at 20 months. It was better to compare 1st–3rd graders at 8 months with 2nd–4th graders at 20 months in order to discussed factors affected traumatic symptoms of children who survived huge disaster over time. According previous study using PTSSC-15, the score of PTSSC-15 increased with age of children [7]. Therefore, if we compared 1st–3rd graders at 8 months with 2nd–4th graders at 20 months, we could not determine whether the improvement because of age, or because of the environment change. We had to try another designed study to compare the same age group at 8 months and at 20 months according to the fact that those examined at 8 months were a year older at 20 months. This study was merely to observe the change over time in the trauma symptoms of children living in a tremendous disaster area.

This study was a survey using a self-completion questionnaire conducted in only one district in Japan. It is impossible to calculate the morbidity of PTSD in children after the 2011 Japanese Earthquake and Tsunami based on the results of the survey. Furthermore, this survey did not follow the cause of each individual's traumatic symptom. This study only shows improvement in trauma symptoms of children in Ishinomaki City. Therefore, this study is insufficient as an epidemiological survey and cohort study for psychiatric diagnosis. Examinations by child psychiatrists using operational diagnostic criteria and structured interviews are necessary for accurate psychiatric diagnosis. Moreover, the results of this study on children in Ishinomaki City do not reflect all characteristics of children affected by the 2011 Japanese Earthquake and Tsunami.

## Conclusions

This study elucidated that traumatic symptoms and daily life activity of children who survived the earthquake and tsunami improved with time. It is important to not only evaluate the traumatic symptoms using a self-completion questionnaire but also confirm specific information regarding the function of family support for children, such as ensuring that the child has breakfast.
